# Clinical Results of Holmium-166 Radioembolization with Personalized Dosimetry for the Treatment of Hepatocellular Carcinoma

**DOI:** 10.3390/jpm14070747

**Published:** 2024-07-14

**Authors:** Christian Kühnel, Alexander Köhler, Tim Brachwitz, Philipp Seifert, Falk Gühne, René Aschenbach, Robert Freudenberg, Martin Freesmeyer, Robert Drescher

**Affiliations:** 1 Clinic of Nuclear Medicine, University Hospital Jena, Am Klinikum 1, 07747 Jena, Germany; christian.kuehnel@med.uni-jena.de (C.K.);; 2 Institute of Diagnostic and Interventional Radiology, Jena University Hospital, Am Klinikum 1, 07747 Jena, Germany; 3 Department of Nuclear Medicine, University Hospital Carl Gustav Carus, Technical University Dresden, 01307 Dresden, Germany

**Keywords:** HCC, microspheres, radioembolization, holmium-166, dosimetry, holmium platform, TARE, SIRT

## Abstract

Transarterial radioembolization (TARE) with ^166^Ho-loaded microspheres is an established locoregional treatment for hepatocellular carcinoma (HCC), introduced in 2010. This study evaluates the clinical outcome of patients with HCC who underwent ^166^Ho-TARE with personalized dosimetry. Twenty-seven patients with 36 TARE procedures were analyzed. Treatment planning, execution, and evaluation was possible without complications in all cases. At the 3-month follow-up, disease control in the treated liver was achieved in 81.8% of patients (complete remission, partial remission, and stable disease in 36.4%, 31.8%, and 13.6%, respectively). The median overall survival (OS) was 17.2 months, and progression-free survival (PFS) in the treated liver was 11 months. Statistically significant positive correlations were observed between the achieved radiation dose for the tumor and both PFS (r = 0.62, *p* < 0.05) and OS (r = 0.48, *p* < 0.05), suggesting a direct dose–response relationship. The calculated achieved dose was 8.25 Gy lower than the planned dose, with relevant variance between planned and achieved doses in individual cases. These results confirm the efficacy of the ^166^Ho-TARE holmium platform and underscore the potential of voxel-based, personalized dosimetry to improve clinical outcomes.

## 1. Introduction

Transarterial radioembolization (TARE) with holmium-166-loaded microspheres has emerged as a treatment option for liver malignancies [1]. Advances in dosimetric techniques have influenced treatment planning, emphasizing the necessity for personalized dosing strategies to maximize therapeutic efficacy while minimizing the risk to healthy liver tissue [2,3].

In the context of TARE, personalized dosimetry means a voxel-based evaluation of the distribution of radioactivity in the liver after TARE planning and treatment procedures, allowing for the prediction of tumor and non-tumor tissue doses [4,5]. By customizing the dose based on individual patient parameters, clinicians can improve the accuracy of treatment delivery, enhance the safety profile of the procedure, and, potentially, achieve better clinical outcomes [6]. Studies have demonstrated that personalized dosimetry is particularly advantageous in cases involving complex tumor geometry or heterogeneous tissue composition, where traditional volume-based methods may be inadequate [7]. This tailored approach not only enhances the predictability and safety of TARE, but also enables the identification of patients who are most likely to benefit from the treatment, thereby optimizing the overall therapeutic success [8,9,10]. A significant dose–response relationship in patients with metastasizing colorectal cancer (mCRC) who underwent ^166^Ho radioembolization has been shown [11,12].

The “Holmium Platform” (Terumo Europe NV, Leuven, Belgium) represents a comprehensive approach to the planning, delivery, and assessment of TARE. It comprises ^166^Ho-loaded poly-L-lactid acid (PLLA) planning microspheres (QuiremScout), a software package for voxel-based dosimetry (Q-Suite), and ^166^Ho-loaded PLLA treatment microspheres (QuiremSpheres). The number and specific activity of microspheres used for a planning procedure are lower than those used for treatment, namely a maximum of 3 million microspheres and 80 Bq/sphere. Q-Suite can process imaging data obtained from SPECT/CT and MRI. Volumes of interest (VOIs) are defined for target and non-target tissues, and a voxel-based dosimetry planning and post-therapeutic evaluation is performed. The planning scintigraphy can also be performed with technetium-99m-labeled albumin microspheres (humane serum albumin, ^99m^Tc-HSA) or macromolecules (macroagglutinated albumin, ^99m^Tc-MAA).

The prerequisite for a favorable outcome after radioembolization is a sufficiently high dose being delivered to the tumor while keeping the dose delivered to the non-tumor liver tissue reasonably low to avoid liver function deterioration. It is expected that personalized dose planning will be superior to volume-based dose planning alone, while also identifying patients who are not suitable for radioembolization [13,14]. This study describes the clinical results of the first group of patients to undergo personalized ^166^Ho-TARE in our institution.

## 2. Materials and Methods

### 2.1. Inclusion and Exclusion Criteria

Patients with HCC who were scheduled for TARE treatment with ^166^Ho-loaded microspheres were included in the study. The treatment was established by a multidisciplinary tumor board specialized in hepatic, pancreatic, and biliary diseases. Initial imaging included liver-centered multiphase CT and/or MRI, as well as thoracic–abdominal CT for staging purposes. Patient histories and pre-TARE oncological treatments were analyzed. Exclusion criteria included a performance status (PS) of 2 or higher and a liver tumor load >70%. A Child–Pugh score of <8 was considered to be preferable, but exceptions were allowed if only a part of the liver was to be treated. The study was conducted in adherence with the Declaration of Helsinki and approved by the hospital’s ethics committee (reg. no. 2022–2652).

### 2.2. The ^166^Ho-TARE Platform

TARE planning and treatment involved comprehensive angiographic imaging of the hepatic vasculature (Artis zeego Q, Siemens Healthineers, Erlangen, Germany). For TARE planning, a hepatic angiogram was performed to determine treatment position(s) in the hepatic arterial vasculature supplying the desired liver target area. Arteries distal to this catheter position(s) posing a risk of extrahepatic microsphere deposition were coil-embolized. Then, 80–170 MBq ^166^Ho-loaded scout microspheres (QuiremScout) per treatment position were injected using the dedicated Quirem delivery system. In selected cases, the procedures were performed using 150–200 MBq ^99m^Tc-labeled human serum albumin microspheres (HSA B20; ROTOP, Dresden, Germany). SPECT/CT images were acquired to determine microsphere distribution in the liver, surrounding tissues, and lung (Symbia Intevo Bold scanner, Siemens Healthineers, Erlangen, Germany; energy windows at 81 keV/15% width and upper scatter at 118 keV/12% width for ^166^Ho, and at 140 keV/15% width including 15% lower scatter window for ^99m^Tc, respectively).

Dose planning and calculation of the prescribed activities per treatment position were conducted with the Q-Suite software package (Q-Suite v2.1, Quirem Medical B.V., Deventer, the Netherlands) by a senior nuclear medicine expert and a medical physics expert. Volumes of interest (VOIs) for the whole liver, target liver, and tumor tissue were drawn manually. Lung segmentation was performed automatically. Target liver, tumor, and non-tumor target liver volumes, activity distribution in the tumor and non-tumor tissue, lung shunt fraction, and expected voxel-based local doses were calculated.

During the TARE treatment, microcatheters (Progreat 2.7 F/130 cm, Terumo Europe NV, Leuven, Belgium) were precisely positioned as planned, and ^166^Ho-loaded microspheres were injected in adherence with the manufacturer’s recommendations [15,16]. Vascular closure devices were used after all procedures. Postprocedural SPECT/CT imaging was performed 42–48 h after the TARE treatment to verify the microsphere distribution and to check for extrahepatic deposits. Dose distribution was analyzed with the Q-Suite software package and compared with planned values. Patients were discharged 48 h after the TARE treatment, in line with local radiation protection regulations.

### 2.3. Residual and Excreted Activities

To compensate for the anticipated residual activity in the application systems, an additional 5% of activity was ordered for each treatment. The actual residual activities in the delivery systems were measured to determine the actual activity of ^166^Ho deposited in the patient per treatment procedure. The urine excretion of the microsphere activity within the first 48 h after the TARE treatment procedures was measured while the patients stayed in the nuclear medicine ward.

### 2.4. Follow-up

Following microsphere administration, patients were accommodated in a nuclear medicine unit for a duration of 24 h for TARE planning or 48 h for the TARE treatment. Any adverse events that arose during this period requiring medical intervention, such as medications, were documented as periprocedural incidents. The classification of all adverse events adhered to the Common Terminology Criteria for Adverse Events (CTCAE). The follow-up period for patients extended to at least 12 months after the initial ^166^Ho-TARE procedure or until the occurrence of their demise. The imaging follow-up for an initial assessment of the response was conducted three months following the finalization of the TARE procedures, which acted as the foundation for devising further treatment strategies. The follow-up phase ended 12 months after the treatment of the final patient.

### 2.5. Outcome Evaluation and Statistics

Overall survival (OS) was defined as the interval between either the initial diagnosis of HCC or the commencement of the first ^166^Ho-TARE treatment and the date of death or the end of the follow-up period for surviving patients. Progression-free survival (PFS) was defined as the interval between the initiation of the first ^166^Ho-TARE treatment and the earliest documented disease progression on imaging, mortality, or the conclusion of the follow-up period.

Treatment response assessment on imaging was based on the modified response evaluation criteria in solid tumors (mRECIST) criteria, categorizing outcomes into complete response (CR), partial response (PR), stable disease (SD), and progressive disease (PD). Disease control was defined by achieving CR, PR, or SD statuses.

Statistical analyses were performed using the open source R programming language (RStudio v 2023.12.1; Posit PBC, Boston, MA, USA). Descriptive statistics initially characterized the dataset. The Pearson’s method was employed to detect correlations between variables. The Kaplan–Meier method was employed to analyze OS, and confidence intervals were used to estimate the precision of survival times. Bland–Altman plots were utilized to evaluate the agreement among dosimetry data acquired during the planning and treatment, allowing for the detection and quantification of systematic discrepancies between these steps. A *p* value of <0.05 was considered to be significant.

## 3. Results

### 3.1. Patient Characteristics and Status before TARE

From April 2021 to February 2023, 27 patients underwent 36 ^166^Ho-TARE procedures. The majority of the patients were male and had underlying liver cirrhosis, an HCC stage of II or IIA (multifocal liver tumor, no metastases), and preserved liver function (Table 1).

The procedure ^166^Ho-TARE was performed as a bridging-to-transplant treatment in 11 patients and as a palliative treatment in 16 patients (40.7% and 59.3%, respectively). For 19 patients, ^166^Ho-TARE was the first-line treatment, with a mean time interval between HCC diagnosis and TARE of 2.4 ± 1.5 months (median of 2.0 months; range of 0.6–6.3 months). Six patients had a progressive disease after the transarterial chemoembolization (TACE). In the two patients with previous ^90^Y-TARE, the ^166^Ho-TARE was performed to treat new HCC lesions in the contralateral liver lobe (interval of 5 months) and to treat tumor recurrence in the same lobe (interval of 40 months). One patient underwent systemic therapy. This 61-year-old man with HCC stage IIIA at diagnosis was initially considered to be ineligible for surgery and locoregional treatment. Over 2 ½ years, he received lenvatinib, sorafenib, and cabozantinib, the latter achieving remission for 13 months. The ^166^Ho-TARE became appropriate when multifocal progression in the right liver lobe with portal vein infiltration was detected.

Four patients with BCLC score C and one patient with BCLC score D underwent ^166^Ho-TARE as part of individual treatment strategies. Two patients were BCLC C due to a macrovascular invasion by the tumor, and two patients had a PS 1 combined with CPS A6 and B8, respectively. The BCLC D patient (CPS C11, PS 1) was evaluated for LTx, and a bridging therapy was strongly recommended by the tumor board in view of an expected low proportion of treated liver.

### 3.2. ^166^Ho-TARE Interventional Procedures and Dosimetry Evaluations

**Figure 1:**
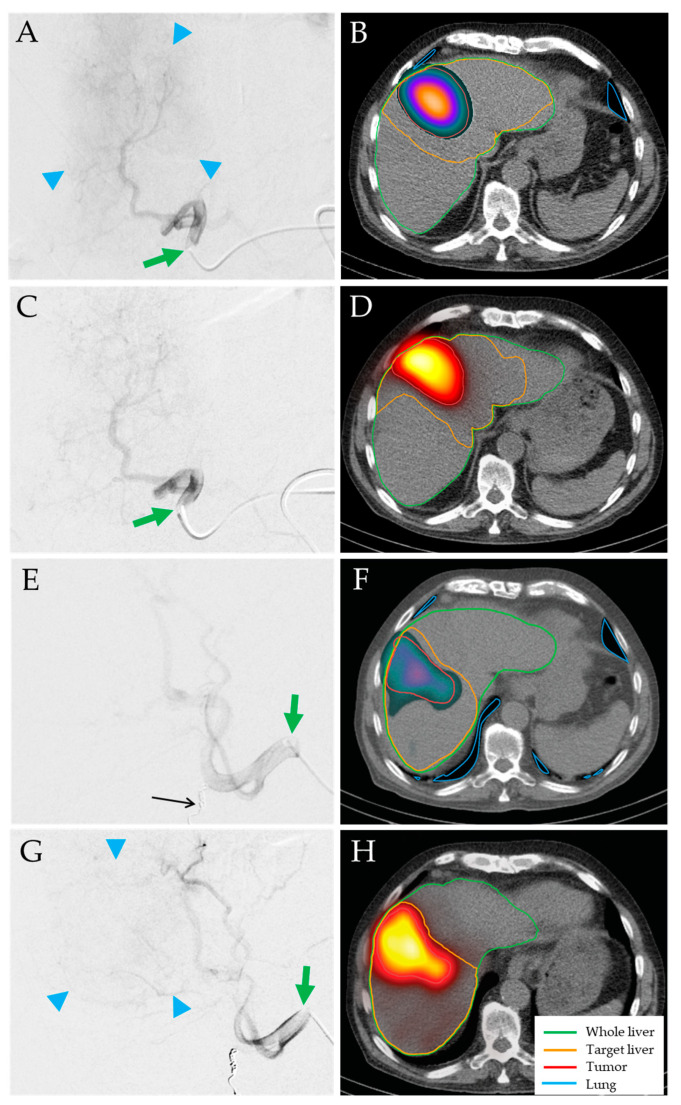
Bilobar sequential TARE treatment of an 83-year-old man with HCC stage IB (singular 9.5 cm lesion involving segments IVa, IVb, V, and VIII). Left lobar planning (**A**,**B**) and treatment (**C**,**D**) with QuiremScout and QuiremSpheres microspheres from the left hepatic artery (green arrows). Planned tumor/healthy target doses of 164 Gy/19 Gy, prescribed activity of 3.9 GBq ^166^Ho. Achieved doses of 208 Gy (+27%) and 19 Gy (=). Right lobar planning (**E**,**F**) and treatment (**G**,**H**) from the right hepatic artery (green arrows) after coil embolization of the cystic artery (**E**, black arrow). Planned tumor/healthy target doses of 137 Gy/38 Gy, prescribed activity of 3.1 GBq ^166^Ho. Achieved doses of 171 Gy (+25%) and 37 Gy (−3%). Strong tumor blushes during both injections confirmed hypervascularity of the tumor (**A**,**G**, blue arrowheads), resulting in high tumor-to-healthy-target ratios for both treatments.

Details of the procedures and dosimetry evaluations are shown in Table 2. All procedures, including planning and treatment, were performed without complications. Seven patients (25.9%) were treated with a bilobar sequential approach (Figure 1). One patient underwent sequential ^166^Ho-TARE of the right liver lobe and segment IV, followed by a third ^166^Ho-TARE of the left liver lobe 6 months later to treat residual vital tumor (no progression). No periprocedural adverse events requiring further intervention or prolonging the hospital stay occurred. Mild-to-moderate upper abdominal pain occurring after nine TARE treatment procedures (25%) was treated symptomatically with oral metamizole for a maximum of two days.

Processing of the SPECT/CT images in Q-Suite was possible in all cases, including nine planning procedures which were performed with ^99m^Tc-HSA B20 microspheres instead of QuiremScout microspheres. In these cases, the decision to use ^166^Ho-loaded microspheres for treatment was made only after the planning procedure.

The mean prescribed activity of 3.4 GBq ^166^Ho was 6.7% lower than the activities which would have been prescribed when using the MIRD-based formula A [MBq] = 3781 [MBq/kg] × liver weight [kg] (target liver absorbed dose 60 Gy; mean 3.2 ± 2.2 GBq, median of 2.8 GBq; range of 0.56–8.8 GBq).

The residual activity in the administration systems after TARE treatments was 5.8 ± 1.2% (median of 4.8%; range of 3.3–8.9%). The mean excreted amount of activity after TARE planning and treatment procedures was 0.0038 ± 0.0025% (median of 0.0036%; range of 0.0004–0.0096%) and 0.0061 ± 0.0037% (median of 0.0053; range of 0.0012–0.0184%), respectively.

**Figure 2:**
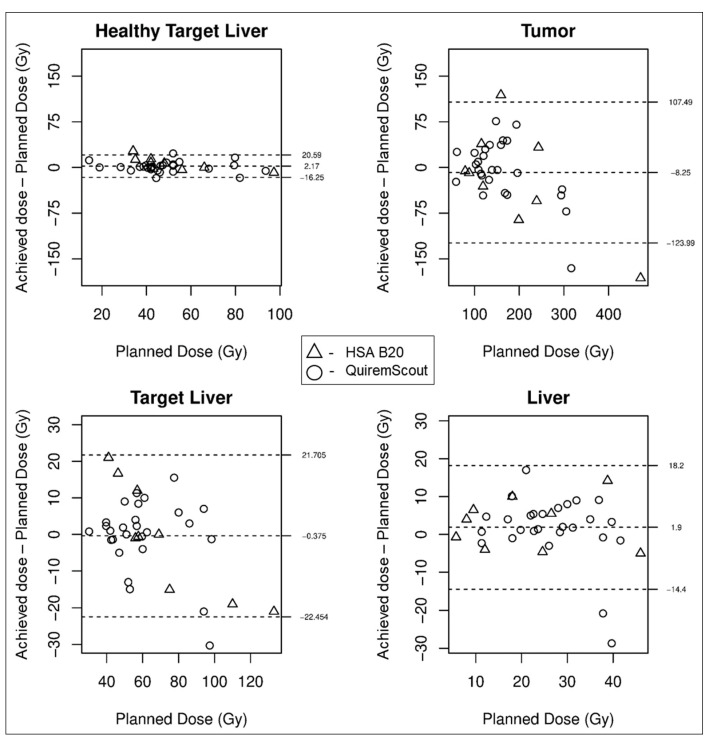
Correlations between planned and achieved doses in 36 TARE procedures (dashed lines: mean, upper/lower limits of agreement).

**Figure 3:**
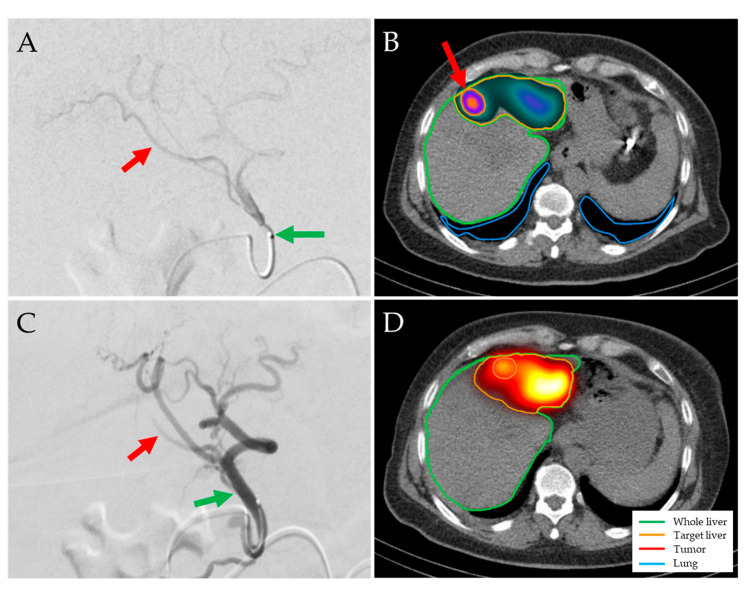
Discrepancy between planning and treatment procedures in a 65-year-old woman with HCC stage IIIA. QuiremScout microspheres were injected into the left hepatic artery (**A**, green arrow). SPECT/CT showed satisfactory activity deposition in the tumor in the liver segment IVa (**B**, red arrow; planned tumor/healthy target doses of 164 Gy/55 Gy). A total of 0.8 GBq QuiremSpheres microspheres were injected (**C**). Post-therapeutic imaging showed a different microsphere distribution with higher activity in non-tumor tissue (**D**, mean tumor/healthy target doses of 86 Gy/57 Gy), possibly due to a slightly more distal catheter position and a segment IV branch (**A**,**C**, red arrows) arising distally but opposite to the microcatheter tip (**C**, green arrow).

Planned and achieved doses delivered to the tumor and healthy liver correlated strongly (r = 0.72 and r = 0.87, respectively), both for planning procedures performed with QuiremScout (r = 0.73 and 0.90) and ^99m^Tc-has B20 (r = 0.72 and 0.85), respectively. On average, the calculated achieved dose delivered to the tumoral tissue was 8.25 Gy lower than the planned dose, whereas mean doses delivered to the whole liver, target liver, and healthy target liver were nearly equal (Figure 2). The variance between planned and achieved doses was higher for the tumoral tissue than for the healthy target liver (upper/lower limits of agreement: 107.49/−123.99 Gy and 20.59/−16.25 Gy, respectively) (Figure 2 and Figure 3). No systematic correlations with tumor size, planning product (^99m^Tc-HSA B20 or QuiremScout), or the amount of injected activity (and, therefore, number of microspheres) were found.

The highest dose delivered to the tumor, i.e., 472 Gy, was planned for a patient with a single HCC lesion in the right liver lobe to treat a small target volume of 123 mL. A dose of 290 Gy delivered to the tumor was reached (−38.5%), leading to complete tumor remission. The lowest dose delivered to the tumor, i.e., 59 Gy, was planned for a bridging-to-transplant, right-lobe treatment in a patient with low tumor load in the target liver and an unfavorable tumor-to-healthy-liver ratio. Planning was conducted with the aim of delivering 60 Gy to the target liver. Postprocedural dosimetry showed a tumor dose of only 35 Gy (−40.7%) due to a higher dose in the large healthy liver volume (+8.3%). A follow-up showed stable disease. 

### 3.3. Treatments and Clinical Outcome after ^166^Ho-TARE

**Figure 4:**
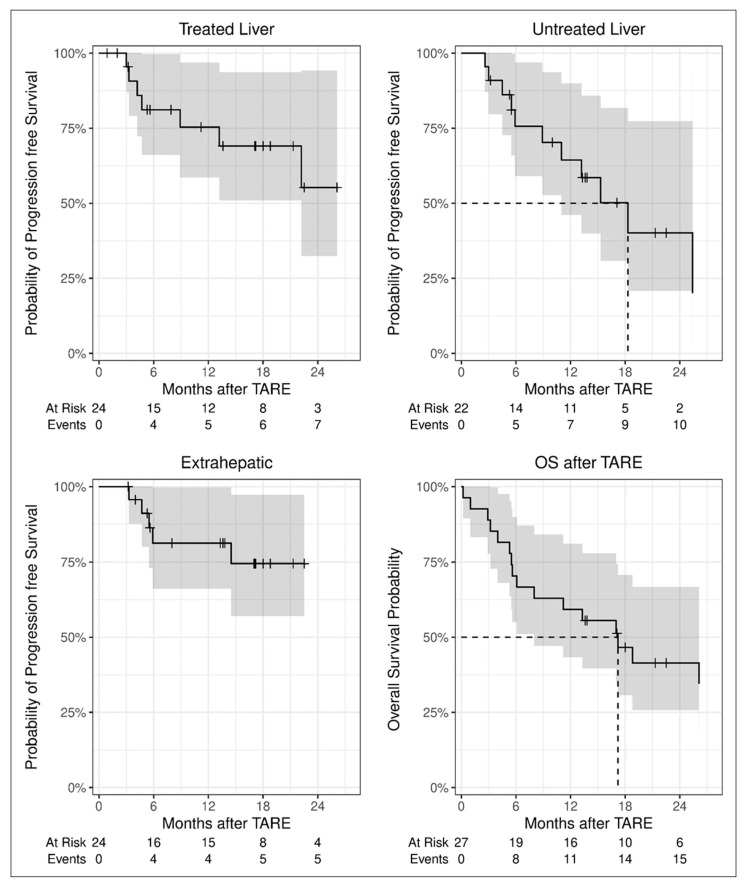
Progression-free and overall survival after ^166^Ho-TARE. Progression in the liver segments treated by ^166^Ho-TARE was detected in seven of 24 patients who underwent an imaging follow-up. Progression in the untreated liver, equivalent to the appearance of new HCC lesions, occurred earlier and more frequently.

The mean follow-up interval for the 27 patients was 13.8 months (range of 0.2–32.4 months). During the course of the study, tumor progression was observed in 14 patients (51.9%). Seventeen patients (63.0%) died, of whom 11 patients had tumor progression. Survival analyses showed an estimated median PFS in the treated liver above 22.2 months, resulting in estimated progression-free rates of 75.3% and 55.2% at 12 months and 24 months, respectively, and a median overall survival of 17.2 months (Table 3, Figure 4).

**Figure 5:**
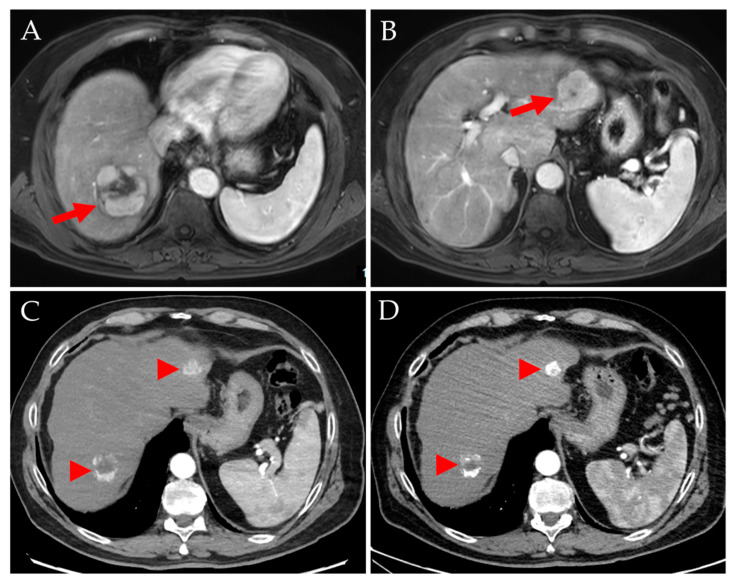
Complete remission after bilobar sequential TARE treatment of a 68-year-old man with HCC stage IIIA, MR imaging (**A**,**B**; T1-weighted fat-saturated sequence with contrast enhancement) shows multiple hypervascular lesions with a diameter of up to 5.3 cm (**A**, arrow). A follow-up CT scan 3 months after TARE revealed complete remission of all lesions according to the mRECIST criteria (**C**, arrowheads), the largest lesion measuring 2.8 cm. Further shrinkage was observed after another 4 months (**D**). The hyperdense areas in the regions of highest microsphere density are a typical finding after ^166^Ho-TARE.

A three-month follow-up could be performed in 22 of 27 patients (81.5%; 2 LTx and three deaths prior to imaging). It showed disease control in the treated liver in 18 patients (81.8%): CR in eight patients (36.4%, Figure 5), PR in seven patients (31.8%), and SD in three patients (13.6%). PD in the treated liver was detected in four patients (18.2%). New lesions in untreated liver segments were seen in two patients, new lymph node metastases were observed in one patient.

In ten patients, the PFS of the treated liver was longer than 12 months. In two of these, tumor progression was detected on imaging. In the other patients, no progression occurred until the end of the follow-up (including the case shown in Figure 1). The 10 patients were among the 12 patients with the longest overall survival. Regarding findings during the 3-month follow-up, the group included six of eight patients with initial CR, four of seven patients with initial PR, two of three patients with initial SD, but no patient with initial PD despite further treatments.

Fourteen patients (51.9%) underwent further treatment of residual viable tumor in the liver, local tumor progression, or extrahepatic spread: five patients (18.5%) received systemic therapy, four patients (1.1%) each underwent LTx, ^90^Y-TARE, and TACE, and one patient (3.7%) underwent percutaneous radiation. For 12 patients (44.4%), ^166^Ho-TARE was the sole treatment during the course of their disease until the end of the follow-up. Of 11 patients who underwent ^166^Ho-TARE as a bridging-to-transplant treatment, four (36.4%) proceeded to LTx, which was performed 0.9, 2.0, 7.9, and 29.2 months after ^166^Ho-TARE. No tumor recurrence after LTx was detected. 

Five patients (18.5%) developed new extrahepatic metastases, with a mean interval after ^166^Ho-TARE of 6.8 ± 3.9 months (median of 5.5 months; range of 3.3–14.5 months), including lymph node (three patients), lung (three patients), and adrenal (one patient) lesions. In three patients, systemic therapy was initiated (atezolizumab/bevacizumab). Four patients with metastases died during the follow-up, with a median overall survival of 8.7 months.

Statistical analyses for the whole cohort showed weak-to-moderate negative correlations between clinical scores and hepatic PFS in the treated liver (hPFS-t; r = −0.31 to r = −0.56; statistically significant for BCLC score, HCC stage, and ALBI score/grade), and OS (r = −0.26 to r = −0.53, statistically significant for BCLC score, and ALBI score/grade). The comparison of patients with ALBI grades 1, 2, and 3 showed a median overall survival of 29.1, 11.2, and 2.8 months, respectively. Patients with BCLC scores 0, A, and B had a median overall survival of 26.1 months, while those with scores C and D exhibited a median overall survival of 5.3 months.

**Figure 6:**
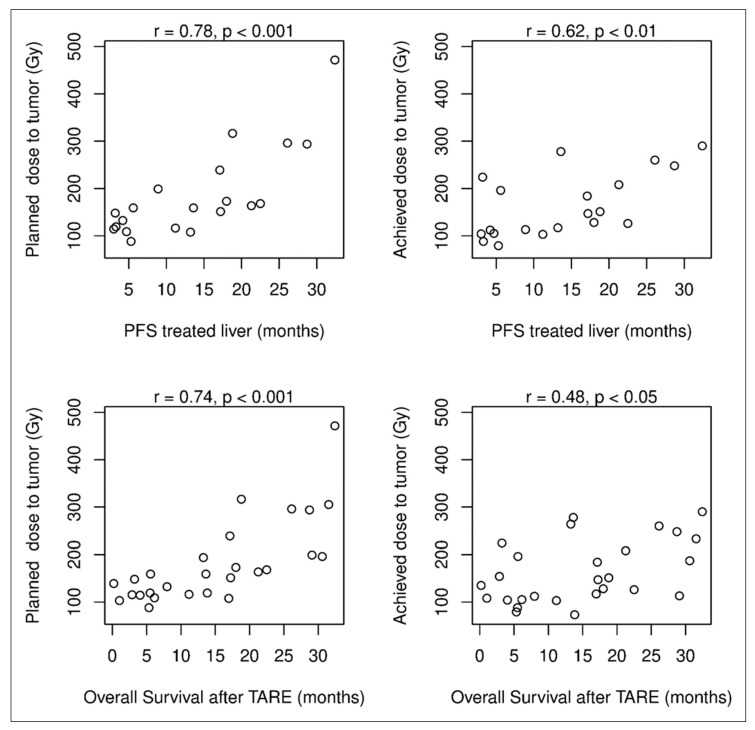
Correlations between dose delivered to the tumor and PFS in the treated liver (upper row; patients with LTx excluded) and between dose delivered to the tumor and OS (lower row) suggest a positive dose–response relationship.

Comparisons between dosimetry data and outcome showed strong positive correlations between planned tumor doses and hepatic PFS in the treated liver, on one hand, and OS, on the other (Figure 6). Moderate positive correlations were found between achieved tumor doses and hepatic PFS in the treated liver, on one hand, and OS, on the other. No correlations were found among planned/achieved healthy liver doses, the proportion(s) of treated liver, and hPFS-t/OS, regardless of the number of ^166^Ho-TARE procedures. A positive relationship was found between hPFS-t and OS in the five patients who had progression in the treated liver and died during the follow-up (*p* = 0.69, not significant).

Eight patients in the study cohort (29.6%) died within six months of ^166^Ho-TARE. All of them underwent one ^166^Ho-TARE procedure only (four right liver lobe, two left liver lobe, one segment II, one segment IV). Four patients (14.8%) died due to causes unrelated to ^166^Ho-TARE: pulmonary embolism (1.0 months after ^166^Ho-TARE), complications during emergency surgery for an incarcerated hernia (2.9 months), cerebral infarction (5.5 months), and gastroenteric hemorrhage (5.6 months; likely as a complication of liver cirrhosis, as no extrahepatic activity deposition was detected after ^166^Ho-TARE). Four patients died due to liver-related causes:

- A 58-year-old man with HCC stage II and severe liver cirrhosis (BCLC D, ALBI grade 3, Child–Pugh score C11, moderate ascites, bridging-to-transplant ^166^Ho-TARE of liver segment II, target 14% of whole liver, healthy target/tumor doses: 59 Gy/119 Gy). The patient was discharged as planned, but readmitted to hospital with acute or chronic liver failure (ACLF) grade 3, pneumonia, and sepsis. The Child–Pugh score deteriorated to C13. The patient died 6 days after ^166^Ho-TARE.
- A 74-year-old man with HCC stage II and liver cirrhosis (BCLC B, ALBI grade 2, Child–Pugh score A6, no ascites, palliative ^166^Ho-TARE of left liver lobe, 43% of whole liver, healthy target/tumor doses: 49 Gy/148 Gy). The follow-up at three months showed PR of the tumor, but moderate ascites (Child–Pugh score B8). The liver function deteriorated rapidly. The patient died 3.2 months after ^166^Ho-TARE.
- A 69-year-old man with HCC stage IIIB and liver cirrhosis (BCLC B, ALBI grade 2, Child–Pugh score B7, moderate ascites, palliative ^166^Ho-TARE of right liver lobe, target 55% of whole liver, healthy target/tumor doses: 41 Gy/114 Gy). The follow-up at three months showed PD in both liver lobes with macrovascular involvement and a Child–Pugh score of B8. The patient died 4.0 months after ^166^Ho-TARE.
- A 65-year-old man with HCC stage II in the liver segment IV, maximum diameter 19 cm compressing the portal vein and liver veins, no liver cirrhosis (BCLC C, ALBI grade 3, Child–Pugh score B8, no ascites, palliative ^166^Ho-TARE of the liver segment IV, 28% of whole liver, healthy target/tumor doses: 42 Gy/88 Gy; tumor not completely perfused). The follow-up at three months showed PR of the tumor and tumor progression in adjacent liver segments. Four months after ^166^Ho-TARE, the liver function deteriorated rapidly (Child–Pugh score C13). The patient died 5.6 months after ^166^Ho-TARE.

## 4. Discussion

### 4.1. Patient Characteristics and Status before TARE

The patients included in this study reflect the clinical reality of a tertiary hospital with a focus on liver surgery and hepatology, as well as a liver transplantation center, explaining the relatively high proportion of bridging-to-transplant treatments. Most of the patients treated here have hepatocellular carcinoma (HCC) secondary to liver cirrhosis. As in most European countries, alcohol is one of the major risk factors [17].

In the treatment sequence for HCC, in the absence of extrahepatic metastases, locoregional treatments are preferred over a systemic therapy. When discussing the appropriateness of TARE, alternatives including surgery, TACE, or stereotactic radiotherapy are also assessed. Since most HCC cases are diagnosed at a multifocal, non-metastatic stage (AJCC stage II/III, BCLC score A/B), TARE is often used as a first-line therapy [18]. In recent years, TARE has also been recommended in our center for patients with fewer than five HCC lesions instead of TACE. If TACE proves unfeasible during the angiography, a TARE planning examination can be conducted in the same session. In individual cases, a tumor board recommendation for TARE may be made even for patients with significantly impaired liver function (five patients with BCLC score C or D in this study) if the therapy is deemed important, such as bridging to liver transplantation. The most common prior therapy in this study was TACE, after which new multifocal lesions had emerged. Previous ^90^Y- or ^166^Ho-TARE is not considered to be a contraindication if the liver function is stable, but, if the same liver lobe or segments are targeted, the previous regional radiation exposure of the liver has to be considered during TARE planning [19].

### 4.2. ^166^Ho-TARE Interventional Procedures and Dosimetric Evaluations

In the absence of comparative studies, the decision of which type of microspheres to use was made at the discretion of the nuclear medicine specialists and radiologists performing the treatment. The primary difference between ^166^Ho- and ^90^Y-microspheres is the shorter half-life and higher local dose rate of the latter. Therefore, the decision is based on personal experience and on the patient’s individual situation, with a tendency to use ^166^Ho to treat fast-progressing tumors. If this was made beforehand, the holmium platform was utilized for patients undergoing ^166^Ho-TARE, with TARE planning conducted using the dedicated ^166^Ho-PLLA planning microspheres (QuiremScout). The advantage of this approach is that the microspheres used for planning and therapy differ only in terms of activity per sphere, but not in size or density. Compared to ^99m^Tc-MAA, a more accurate prediction of intrahepatic distribution can be achieved [20]. A disadvantage is that QuiremScout must be ordered for a specific day and cannot be radioactively labeled on site. The alternative, in our institution, is HSA B20, microspheres of albumin with a diameter of 10–30 µm which can be labeled with technetium-99m and are eliminated from the capillaries with a biological half-life of 7.2 h [21]. Given that they are microspheres and not irregular macromolecules, the predictive accuracy of HSA may be superior to that of MAA, although this has not yet been investigated. The only comparative study has demonstrated that HSA is more resistant to degradation over time, thus enabling reliable SPECT imaging at later time points [22].

No periprocedural complications requiring intervention or prolongation of hospitalization occurred with respect to the patients in the study. The upper abdominal pain observed in one-quarter of the patients responded well to metamizole. Throughout our experience with TARE treatments, we have observed that pain is most commonly experienced after TARE with ^90^Y resin microspheres (due to their high number and, therefore, embolic effect), and only rarely after TARE with ^90^Y glass microspheres. This postembolic pain responds poorly to traditional analgesics (metamizole, paracetamol, ibuprofen), but subsides within 1–2 days.

In general, a lower level of activity was prescribed using voxel-based dosimetry for TARE planning compared to the MIRD formula, which typically targets a dose of 60 Gy to be delivered to the liver volume. This personalized approach allows for greater safety and flexibility throughout the treatment. In cases with a high tumor-to-healthy-liver ratio, a sufficient dose delivered to the tumor could be reached with less prescribed activity. In patients where only a small portion of the liver was treated and/or in patients with a high tumor burden within the target volume, dose escalation was possible.

Planned and achieved doses delivered to tumor and healthy target liver correlated strongly, but there was notable variance in relation to individual procedures. A limitation of accurate planning and evaluation is the user dependency when drawing the VOIs, as the volume of the target volume in particular depends on the windowing of the SPECT, which is, at least, partially subjective. It was noted that very high planned tumor doses were not achieved. This discrepancy may be attributed to a technical limitation, as the post-therapeutic SPECT/CT was not performed later than 48 h after the TARE treatment (i.e., less than two half-lives of ^166^Ho), due to organizational constraints. Possibly, the validity of the SPECT quantification was lower than that of the planning SPECT due to a relevant camera dead time when high ^166^Ho activities are still present [23].

The degree of agreement regarding the dose delivered to the healthy liver was slightly greater than that regarding the dose delivered to the tumor (r = 0.87 and r = 0.72, respectively). This may be attributed to the fact that a discrepancy in microsphere distribution between TARE planning and TARE therapy has a greater impact on a small tumor volume and a less pronounced effect on the larger healthy target liver volume. The agreement between planned and achieved doses delivered to both tumor and healthy target was slightly worse after planning procedures with ^99m^Tc-labelled HSA, with the highest absolute dose deviations in both positive and negative directions (Figure 2, tumor diagram). The differences were not statistically significant.

One potential optimization is the development of PLLA microspheres that can be labeled with technetium-99m on site. Microspheres that can be labeled with gallium-68 could enhance planning accuracy based on PET imaging and warrant consideration for research purposes.

### 4.3. Residual and Excreted Activities

Previous evaluations have shown median residual activities of 4.3% (range of 3.5%–6.9%) remaining in the delivery systems after standardized TARE treatment procedures QuiremSpheres, regardless of the prescribed activity [24]. In this study, an estimated residual waste of 5% was included in the activity calculation. Actual residual activities were similar (median 4.8%), ranging from 3.3% to 8.9%, an amount which does not represent a relevant over- or undertreatment.

The urinary excretions of radioactivity were determined for 48 h after TARE treatments and were very low, not surpassing the regulatory threshold for radioactive amounts in wastewater [25]. These differ depending on national requirements and should be reviewed before performing ^166^Ho-TARE.

### 4.4. Treatments and Clinical Outcome after ^166^Ho-TARE

The selection of the TARE treatment is based on the extent of the malignant disease, liver function, and the patient’s general condition. A diverse range of clinical scoring systems are used [26]. In this study, all used scores (HCC stage, Child–Pugh score, BCLC score, ALBI grade) correlated negatively with PFS and OS, with statistical significance for both criteria for the BCLC score and the ALBI grade. This, again, emphasizes that patients with impaired liver function should undergo TARE with caution, in the absence of alternative treatment and if a surveillance strategy is not feasible, even if only part of the liver is targeted. The pharmaceutical prophylaxis of radiation-induced liver disease should be considered [27].

Patients treated with ^90^Y-TARE at our institution from 2011 to 2020 showed a median overall PFS of 11.0 months and an estimated progression-free rate of 47.5% after 12 months. The median OS was 16.6 months, with an estimated 12-month survival rate of 60.5% [28]. The first 14 patients treated by ^166^Ho-TARE and standard dosimetry had a median PFS in the treated liver of 10.3 months, an overall PFS of 7.3 months, and an OS of 22.1 months [29]. In the present study, the results for PFS in the treated liver were better, with an estimated PFS rate after 12 months of 75.3% (Table 3, Figure 4). The median overall PFS and OS were similar (11.0 months and 17.2 months, respectively). The initial follow-up 3 months after ^166^Ho-TARE showed disease control in 81.8% of patients. At that time, two patients had already undergone LTx and three patients had died, two of them due to causes unrelated to the liver or the HCC. In most cases, patients with the most favorable local response (CR) also had a long PFS in the treated liver. The effect of treatment depth has been described before [30].

Several studies have already shown the advantages of personalized dosimetry, which improves the outcome and reduces hepatotoxicity [2,3,4,8,31,32,33,34]. Sound recommendations for dosimetry planning for different tumor types have been published for ^90^Y resin and ^90^Y glass microspheres [14,35]. For ^166^Ho PLLA microspheres, a significant dose–response relationship and an association between toxicity and parenchymal dose have been found, first in mCRC patients [11,12]. Despite the small patient cohort, this study also showed statistically significant positive correlations between tumor dose and clinical outcome (Figure 6). Correlations were stronger for planned than for achieved tumor doses and also for PFS in the traded liver than for OS. The better prognostic value of the planned tumor dose could lie in the possibly higher accuracy of SPECT imaging in TARE planning. The impact on OS is influenced by additional treatments, and a larger cohort with longer, structured follow-ups would be necessary to achieve reliable results [36].

The PFS in the untreated liver was shorter than in the treated liver, and nearly every fifth patient (18.5%) developed extrahepatic metastases (Table 3). Regarding the untreated liver, this is attributable to small HCC lesions already present but not visible via imaging at the time of TARE, circulating tumor cells, and metastases arising from residual viable tissue in the treated liver. Extrahepatic metastases have similar origins, particularly circulating tumor cells. Further research should analyze whether TARE treatments can decrease the incidence of such cells, because the method may be effective in preventing the invasion of healthy tissue and vasculature, therefore lowering the risk of HCC cell distribution [37,38].

With the impression that disease progression and survival after TARE depends, to a large extent, on prohibiting metastatic lesions in the untreated liver and extrahepatic metastases, combinations with systemic therapies as “TARE-adjuvant treatments” are important. Because TARE is a vascularly targeted therapy, it should have a role in combination with molecular-targeted substances to increase the life expectancy of patients with HCC [39,40]. The underlying hypothesis is that radiation leads to antigen release from tumors, thereby increasing an immunologic response which can be exploited through immunoregulatory systemic drugs [41]. Data regarding ^166^Ho-TARE are still scarce, but, for ^90^Y-TARE, a study comparing combinations with immune checkpoint inhibitors (ICI) and tyrosine kinase inhibitors (TKI) yielded better imaging response rates for the ^90^Y-ICI combination [42]. A consensus paper on combining ^90^Y-TARE with systemic anticancer agents confirmed the safety and potential efficacy of these regimens but called for more clinical trial data for nearly all HCC aspects [43].

For TARE, a negative abscopal effect has also been discussed, where mitogens released from the treated tumors induce tumor growth in the untreated liver [44]. A prospective study evaluating this topic is ongoing [45]. The phenomenon of (positive) abscopal effects, whereby local therapies elicit tumor manifestations beyond the treated region, has been documented in the context of interventional oncology techniques, including radioembolization [46,47].

The quality of life (QoL) of patients undergoing TARE has been evaluated in several studies, demonstrating that these patients exhibit higher QoL scores than those undergoing a systemic treatment, including sorafenib [48]. Patients with liver metastases of colorectal cancer returned to their initial QoL after a temporary decline in the first week after ^166^Ho-TARE [49]. One of the key benefits of TARE is the potential for patients to achieve treatment-free intervals of 3 months or longer, allowing for a recuperative period or for the preparation of subsequent systemic therapies.

### 4.5. Limitations of the Study

The study was monocentric, non-randomized, and observational. The validity of the study results is limited by the small sample size. Inclusion in the study was consecutive based on tumor board recommendations, leading to a highly variable cohort regarding the extent of the malignant disease as well as previous therapies. Local specificities of dosimetric techniques may affect the generalizability of the results. Outcome evaluation was focused on the ^166^Ho-TARE treatments and dosimetric aspects. In-depth subgroup analyses were not possible. Since patients were discharged two days after the TARE treatments and the first study follow-up was conducted after 3 months, intermittent adverse events not requiring intervention or hospitalization may have been underestimated.

## 5. Conclusions

The results of the study indicate that personalized ^166^Ho-TARE is an effective treatment option for patients with HCC, in palliative and bridging-to-transplant settings. The selection of appropriate patients and precise dosimetry planning are critical for therapy success, particularly when considering patients with impaired liver function due to the tumor and liver cirrhosis. New imaging technologies and dosimetry software have enhanced the accuracy of radiation dose calculation. Such advancements allow for a more precise tailoring of the therapy to individual patient anatomy and disease manifestation, potentially improving therapy outcomes. Pre- and post-therapeutic dosimetry is technically feasible and should, in view of the confirmed dose–response relationships, influence clinical decisions with the option to adjust treatment plans based on the actually absorbed dose. Technical improvements are necessary to improve the reliability and user-independence of dosimetry evaluations. These considerations are crucial for maximizing therapy effectiveness and minimizing toxic side effects.

### 5.1. Clinical Relevance

The integration of a planning, treatment, and evaluation platform enhances safety and precision of TARE with ^166^Ho-loaded microspheres and allows for the precise tailoring of the procedure. This has the potential to increase the therapeutic window while minimizing the collateral damage to the surrounding healthy tissues, directly contributing to improved patient management and clinical outcome.

### 5.2. Future Research

Based on the study results, further research should investigate options to improve the accuracy and robustness of dosimetry solutions for TARE. SPECT- and PET-based methods with voxel-based absolute activity quantification should be evaluated, including the introduction of novel TARE simulation products, such as ^99m^Tc-labelled PLLA microspheres and ^68^Ga-labelled PLLA or albumin microspheres.

## Figures and Tables

**Table 1:** Patient and tumor characteristics.

No. of Patients	27
Male/Female	25 (92.6%)/2 (7.4%)
Age (years, at initial diagnosis) *	68 ± 7 (68; 55–82)
Underlying liver disease	
Alcohol-related liver cirrhosis	23 (85.2%)
Nonalcoholic steatohepatitis (NASH)	2 (7.4%)
None	2 (7.4%)
HCC diagnosis to ^166^Ho-TARE (months) *	8.0 ± 16.2 (2.2; 0.6–81.4)
HCC stage	
IA	1 (3.7%)
IB	5 (18.5%)
II	7 (25.9%)
IIIA	10 (37%)
IIIB	2 (7.4%)
IVA	1 (3.7%)
IVB	1 (3.7%)
Child–Pugh stage/score	
A5	18 (66.7%)
A6	4 (14.8%)
B7	3 (11.1%)
B8	1 (3.7%)
C11	1 (3.7%)
Barcelona Clinic Liver Cancer (BCLC) score	
0	1 (3.7%)
A	7 (25.9%)
B	14 (51.9%)
C	4 (14.8%)
D	1 (3.7%)
Albumin–bilirubin (ALBI) grade	
1	12 (44.4%)
2	13 (48.1%)
3	2 (7.4%)
HCC treatments before 166Ho-TARE **	
None	19 (70.4%)
Transarterial chemoembolization (TACE)	6 (22.2%)
90Y-TARE	2 (7.4%)
Systemic therapy	1 (3.7%)

* values are mean ± SD (median, range); ** multiple treatments per patient are possible.

**Table 2:** TARE characteristics.

No. of ^166^Ho-TARE Procedures	36
No. of TARE per patient	
1	19 (52.8%)
2	7 (38.9%)
3	1 (8.3%)
Target: liver region	
Lobar, right	16 (44.4%)
Lobar, left	5 (13.9%)
Segmental	15 (41.7%)
Planning product	
^99m^Tc-HSA B20 microspheres	9 (25.0%)
^166^Ho-PLLA microspheres	27 (75.0%)
Tumor load: proportion of whole liver (%) *	6.2 ± 7.0 (3.8; 0.3–31.9)
Planning *	
Target volume: proportion of whole liver (%)	40.2 ± 18.5 (41.7; 5.8–80.7)
Tumor load: proportion of target volume (%)	16.5 ± 16.8 (11; 1–76.6)
Whole liver dose (Gy)	25.2 ± 10.6 (24.6; 6–46)
Target dose (Gy)	63.4 ± 22.8 (56.9; 30–133)
Healthy target dose (Gy)	49.6 ± 18.5 (45.5; 14–97)
Tumor dose (Gy)	166.3 ± 85.1 (143.5; 59–472)
Lung dose (Gy)	5.3 ± 3.4 (4.9; 1–14)
Prescribed activity (GBq)	3.4 ± 2.0 (3.1; 0.8–8.0)
Treatment *	
Injected activity (GBq)	3.2 ± 1.9 (2.9; 0.8–7.5)
Whole liver dose (Gy)	27.1 ± 11.9 (28.0; 5.0–53.0)
Target dose (Gy)	63.0 ± 19.7 (60; 31–112)
Healthy target dose (Gy)	51.7 ± 18.4 (49.5; 19–96)
Tumor dose (Gy)	158.1 ± 68 (143; 35–290)
Image-based dose reconstruction factor (E-4 MBq/count)	2.5 ± 1.7 (2.1; 1.2–11)

* values are mean ± SD (median, range).

**Table 3:** Progression-free and overall survival analyses.

	Survival		
	Median (Months; 95% CI)	Est. Rate 12 Months after TARE (%; 95% CI)	Est. Rate 24 Months after ^166^Ho-TARE (%; 95% CI)
**Progression-free survival**			
Whole body	11 (5.5, -)	49.7 (31.6, 78.2)	14.5 (3.0, 70.8)
Hepatic, treated liver	- (22.2, -)	75.3 (58.6, 96.9)	55.2 (32.4, 94.2)
Hepatic, untreated liver	18.3 (11, -)	64.4 (46.1, 89.9)	40.1 (20.8, 77.3)
Hepatic, whole liver	11 (5.5, -)	49.7 (31.6, 78.2)	13.6 (2.7, 69.1)
Extrahepatic	- (-, -)	81.2 (66.2, 99.7)	74.5 (57.0, 97.2)
**Overall survival**			
After ^166^Ho-TARE	17.2 (8, -)	59.3 (43.3, 81.0)	41.4 (25.7, 66.7)
After HCC diagnosis	25.6 (15.1, -)	66.7 (51.1, 87.0)	50.1 (33.9, 74.1)

## Data Availability

Additional data sharing is not available due to data protection regulations.
